# Type 1 Diabetes Prone NOD Mice Have Diminished *Cxcr1* mRNA Expression in Polymorphonuclear Neutrophils and CD4+ T Lymphocytes

**DOI:** 10.1371/journal.pone.0134365

**Published:** 2015-07-31

**Authors:** Karine Haurogné, Marija Pavlovic, Hélène Rogniaux, Jean-Marie Bach, Blandine Lieubeau

**Affiliations:** 1 INRA USC1383, IECM, Nantes, France; 2 LUNAM Université, Oniris, EA4644, Nantes, France; 3 INRA UR1268, BIA, BIBS, Nantes, France; University of Leuven, Rega Institute, BELGIUM

## Abstract

In humans, CXCR1 and CXCR2 are two homologous proteins that bind ELR+ chemokines. Both receptors play fundamental roles in neutrophil functions such as migration and reactive oxygen species production. Mouse *Cxcr1* and *Cxcr2* genes are located in an insulin-dependent diabetes genetic susceptibility locus. The non obese diabetic (NOD) mouse is a spontaneous well-described animal model for insulin-dependent type 1 diabetes. In this disease, insulin deficiency results from the destruction of insulin-producing beta cells by autoreactive T lymphocytes. This slow-progressing disease is dependent on both environmental and genetic factors. Here, we report descriptive data about the *Cxcr1* gene in NOD mice. We demonstrate decreased expression of mRNA for *Cxcr1* in neutrophils and CD4+ lymphocytes isolated from NOD mice compared to other strains, related to reduced NOD *Cxcr1* gene promoter activity. Looking for Cxcr1 protein, we next analyze the membrane proteome of murine neutrophils by mass spectrometry. Although Cxcr2 protein is clearly found in murine neutrophils, we did not find evidence of Cxcr1 peptides using this method. Nevertheless, in view of recently-published experimental data obtained in NOD mice, we argue for possible Cxcr1 involvement in type 1 diabetes pathogenesis.

## Introduction

Human *CXCR1* and *CXCR2* genes were characterized a long time ago [1, 2]. CXCR1 protein, like CXCR2, belongs to the large 7-transmembrane (7-TM) domain G-protein coupled receptor family. Both receptors bind ELR+ CXC chemokines. In human, the main ELR+ chemokine is CXCL8, initially called IL-8. While CXCR2 binds all of the seven described ELR+ chemokines, CXCR1 binds CXCL6, in addition to CXCL7 and CXCL8 [3]. In human, CXCR1 and CXCR2 are co-expressed in neutrophil plasma membrane and secretory vesicles, and bear overlapping and independent essential functions. Experimental models have been developed to identify the role of each receptor using *CXCR1* or *CXCR2*-transfected cell lines, and/or selective blocking antibodies on human neutrophils. Both receptors are capable of mediating chemotaxis [4]. Chuntharapai and Kim proposed a two-steps process hypothesis where CXCR2 participates in the initial phase of migration (where CXCL8 concentration is at picomolar level) whereas CXCR1 is mainly involved at inflammation sites (where CXCL8 concentration is at nanomolar concentration) [5]. CXCL8 regulates phospholipase D-mediated superoxide anion production and granule release through CXCR1 signaling [6, 7]. CXCL8-induced CXCR1 activation in neutrophils also promotes respiratory burst and alpha-defensin release, which are key antibacterial host defense functions [8].

Human lymphocytes express CXCR1 as well. Takata *et al*. reported CXCR1 expression in a subset of cytolytic effector CD8+ T cells and suggested that CXCR1 might be important for their homing in various inflammatory and infectious diseases [9, 10]. Also, IL-6-induced CXCR1 expression in regulatory T cells, might cause their migration toward IL-6- and CXCL8-expressing tumors [11].

CXCR1 expression was also reported on cells other than leukocytes [12]. For example, CXCR1 was shown to be involved in colon carcinoma cell migration [13, 14]. Recently, Al-Alwan *et al*. provided the first evidence that hCXCR1, which is not the primary CXCL3 receptor, plays an essential role in CXCL3-induced airway smooth muscle cell (ASMC) migration [15]. Using primary cells, they showed that CXCR1 and CXCR2 are both required for CXCL3-induced normal ASMC migration. In contrast, in asthmatic ASMC, CXCL3-induced migration becomes exclusively CXCR1-dependent, which underlines the significance of CXCR1 in the pathogenesis of airway remodeling associated with asthma. Thus, CXCL8 and its two receptors significantly contribute not only to pathogen clearance but also to tissue injury, fibrosis, angiogenesis and tumorigenesis. Therefore, multiple drugs have been developed for therapeutic use in human in order to target this pathway involved in many disease-associated processes [12].

In mouse, no *Il-8* ortholog gene has ever been identified; yet, Cxcr2 was long called the Il-8 receptor. Until now, five different murine ELR+ chemokines have been described: Cxcl1 (Kc), Cxcl2 (Mip-2), Cxcl3 (Dcip-1), Cxcl5 (Lix/Ena-78, human CXCL6 equivalent) and Cxcl7 (Nap-2, Ppbp).

Mouse *Cxcr1* and *Cxcr2* genes are located into the insulin-dependent diabetes genetic susceptibility locus (*idd*) *idd5*.*2* [16]. Among many genes present in the large *idd5*.*2* region, *Slc11a1*, previously known as *Nramp-1* (“Natural Resistance-Associated Macrophage Protein-1”), is the most compelling candidate gene [17–19]. *Slc11a1* encodes a divalent cation transporter of the Solute Carrier Family, present at phagosomal and lysosomal membranes of macrophages and dendritic cells. The dominant allele mediates protection from certain infections by reducing the ability of some pathogens such as Mycobacterium and Salmonella, to survive in the phagosome. A non-synonymous polymorphism (Gly169 to Asp169) generates a non functional mutant protein, resulting in susceptibility to these intracellular pathogens [20]. C57Bl/6J and Balb/C strains express the mutant protein while the NOD mouse express the functional protein.

The NOD mouse model is a well-described spontaneous model of type 1 diabetes (T1D). T1D is an autoimmune disease, characterized by the selective destruction of insulin-producing cells by autoreactive T cells. Insulin deficiency is the result of a complex long-lasting process which is under the control of both environmental and genetic factors.

Here, we became interested by studying Cxcr1 and Cxcr2 expression in cells isolated from NOD mouse compared to control strains including the NOR mouse. The NOR strain is a MHC-matched diabetes resistant control strain for NOD mouse [21, 22]. Its genome differs from the NOD one, specially through 4 *idd* loci including the *idd5*.*2* one [16]. It never develops spontaneous diabetes, although a few Langerhans islets might become infiltrated with aging. We found decreased expression of *Cxcr1* mRNA in cells isolated from NOD mouse. We showed lower transcription efficiency of the NOD *Cxcr1* promoter in gene reporter experiments, in agreement with multiple polymorphisms described in NOD promoter sequence. Although we could not find Cxcr1 protein in our samples, we next discuss on mouse Cxcr1 protein in view of current literature data.

## Materials and Methods

### Animals

NODShi/Ltj, C57Bl/6J, Balb/C and NOR/Lt mice (Jackson Laboratories, Bar Harbor, MA) were maintained in specific pathogen-free conditions (MICE, Charles River Laboratories, Wilmington, MA) at the Oniris rodent facility (agreement number: 44 266). All care and animal experiments were carried out in strict accordance with French guidelines and under the authority of a license issued by the Direction Départementale des Services Vétérinaires under the applicable law at the time the experiments were performed (B.L. agreement number: B44-69).

### Isolation of bone marrow-derived neutrophils and spleen-derived CD4+ lymphocytes

Bone marrow was flushed out from bones with phosphate-buffered saline (PBS) buffer and Ly-6G+ cells were purified by immunomagnetic sorting (Miltenyi Biotec, Paris, France). Spleen was disaggregated into single cell suspension. After red cells removal using using ammonium chloride lysis buffer, CD4+ cells were purified by immunomagnetic sorting (Miltenyi Biotec). Cell purity was checked by flow cytometry and was always above 95%.

### Real-time quantitative RT-PCR

mRNAs were extracted from 4X10^6^ cells using Dynabeads mRNA DIRECT kit (Fisher Scientific, Illkirch, France) and converted into cDNA using M-MLV Reverse Transcriptase (Promega, Charbonnières, France). Primer (obtained from Eurogentec) sequences were designed using Primer3Plus software (Table 1). Quantitative real-time PCR mRNA analyses (RT-qPCR) were performed on an ABI Prism 7300 Sequence Detection instrument (Applied Biosystems, Courtaboeuf, France) using EvaGreen fluorescence system (Solis BioDyne, Tartu, Estonia) for quantification of amplicons. All cDNA samples were run in duplicate in 96-well optical reaction plates. In each run, control cDNA dilution series were created for each primer pair to establish relative standard curves. Melting curve analysis was performed (65°C-95°C) in order to confirm the presence of unique amplified products. Data were analyzed with the relative quantification standard curve method; gene expression was normalized by using *ß-actin* and *Rpb1* (RNA polymerase II subunit B1) housekeeping genes and GeNorm matrix.

**Table 1:** Primer sequences.

	Forward primer (5’-3’)	Reverse primer (5’-3’)
* m*Cxcr1* genomic regions amplification		
5’region	CTTAACCCAGTCAGCCATTT	CGAAGGGACTGGATGAATAA
	GATCATCTATGGAGGGAAGGA	AGCCACATAACAGGAAAGCA
exon 1	GATCATCTATGGAGGGAAGGA	AGCCACATAACAGGAAAGCA
exon 2	GGGGTGAACTTTGGTTGTC	TCAATCAAGTGGGCTCCTAA
	TTGCTGTTGTGTTGGTCTTC	CCTCCCCAAAGCAGATAGTA
* m*Cxcr1* promoter cloning ^a^		
	FP^b^	RP^b^
	GGGGTACCCCAAAACAAAACAGCAAGCATT	GAAGATCTTGAGGGGACAAGAAACCATTTA
* mRNA quantification^c^		
m *Cxcr1*	CTCCCGCACACAAGGAAC	GCAGCATTCCCGTGATATTT
m *Cxcr2*	GCTCACAAACAGCGTCGTAG	CCACCTTGAATTCTCCCATC
*ß-actin*	TTGCTGACAGGATGCAGAAG	GTACTTGCGCTCAGGAGGAG
*Rpb1*	GACCGAAAGCACATGACTGA	CAATTCAAATCATCGCCAAA

^a^Underlined sequences represent restriction enzyme sites used for cloning.

^b^See Figs 2 and 3 for position in genomic sequence.

^c^GenBank accession numbers are as follows: NM_178241 for *mCxcr1*, NM_009909 for *mCxcr2*, NM_007393 for *ß-actin* and U37500 for *Rpb1*.

### Sequencing of mCXCR1 gene and 5’-flanking region

The Ensembl genome browser (http://www.ensembl.org/) was used to obtain genomic *Cxcr1* sequence [23]. The Genomatix database (http://www.genomatix.de/) allowed the *in silico* identification of the putative *Cxcr1* promoter region [24]. Genomic DNA was extracted from tail fragments by phenol-chloroform protocol. Primers were designed to amplify the exons and intron/exon junctions as well as the 5’ flanking region (Table 1). Amplified fragments were sequenced by GATC Biotech (Mulhouse, France). The NODShi/Ltj mouse *Cxcr1* promoter sequence has been deposited in the Genbank database [25] under number KT156754.

### Plasmid construction

739 bp fragments covering the putative Genomatix mouse *Cxcr1* promoter region (601 bp) were PCR amplified from NOD and NOR mouse genomic DNA using the Phusion DNA polymerase (Ozyme, Saint Quentin en Yvelines, France) and forward primer (FP) and reverse primer (RP) primers (Table 1). Purified PCR products (500ng) were digested by *Kpn*I (10U) and *Bgl*II (10U) (Ozyme) and cloned into the pGL4.10[*luc*2] Luciferase Reporter Vector (Promega). After transformation into competent cells and plasmid amplification, sequences and correct orientation of the inserted promoter regions were checked.

### Cell culture and transient transfections for dual-reporter gene assays

The MH-S mouse alveolar macrophage cell line (American Type Culture Collection, Bethesda, MD) was grown in complete RPMI medium (Eurobio, Courtaboeuf, France).

3X10^5^ MH-S cells were resuspended in 20μl Nucleofector solution (Cell Line 96-well Nucleofector Kit SF; Lonza, Levallois, France) per one well and transfection was performed with Nucleofector device according to manufacturer protocol (Lonza).

To normalize for transfection efficiency, pGL4.10[*luc*2] plasmids bearing either the wild-type *mCxcr1* promoter or the NOD sequence were cotransfected with the pGL4-74[*hRluc*/TK] plasmid, which encodes Renilla luciferase. Negative and positive controls were respectively obtained with empty pGL4.10[*luc*2] vector and pGL3[*luc*/SV40] vector (Firefly luciferase under SV40 large T antigen promoter). Following nucleofection and a 16h-incubation, cells were lysed and luciferase assays were performed using the Dual-Glo Luciferase Assay system (Promega). Luminescence was measured on a Wallac MicroBeta TriLux (Perkin Elmer, Courtaboeuf, France). Values of firefly luciferase activity were normalized to the ones of Renilla luciferase. Each plasmid of interest was transfected into three wells per experiment, and three independent experiments were performed.

### Preparation of cell membrane-enriched proteins

10^8^ NOR mouse neutrophils were suspended in Tris-EDTA buffer (25mM Tris, 1mM EDTA, pH:7.5) containing 7% protease inhibitor cocktail (Sigma-Aldrich) and sonicated by two 15 second-cycles (VibraCell, Bioblock). Unbroken cells, nuclei, and cell debris were removed by centrifugation at 3,000g for 10min. Microsomes were harvested from supernatant by 100,000g ultracentrifugation at 4°C for 1h (Beckman SW41 rotor) and the membrane-enriched pellet was lysed by a 30min incubation at 4°C in RIPA buffer (Sigma-Aldrich) containing 0.5% SDS and 1% protease inhibitor cocktail. Finally, protein lysate was obtained after a 20,000g centrifugation at 4°C for 15min. Protein concentration was determined using the BCA kit (Pierce).

### Gel-based protein purification and in-gel trypsin digestion

20μg to 30μg membrane protein samples were run into SDS polyacrylamide gel comprising a 1cm-high stacking 4% w/v polyacrylamide matrix on top of a 20% w/v polyacrylamide matrix, at 80V until protein samples concentrate at the 4–20% w/v gel interface. After staining with Coomassie blue, protein bands were excised and cut into small pieces. After destaining and washing in a 50:50 mixture of acetonitrile (CH_3_CN) and 50mM ammonium bicarbonate buffer (NH_4_HCO_3_), the proteins were reduced with 10mM dithiothreitrol (DTT, Sigma-Aldrich) for 1h at 57°C and free cysteine residues were alkylated with 55mM iodoacetamide (Sigma-Aldrich) for 45min at room temperature. After further washings of gel slices, proteins were next digested with 12ng/μl trypsin (Promega) in 25mM NH_4_HCO_3_ buffer containing 0.01% ProteaseMAX surfactant (Promega) for 3h30 at 37°C. Once the reaction stopped with 1% formic acid, peptide-containing supernatants were sonicated, vortexed and pooled for concentration by vacuum centrifugation.

### Peptides analysis and protein identification by mass spectrometry

The peptides resulting from in-gel trypsin hydrolysis were analyzed by mass spectrometry (MS) using a LTQ-Orbitrap VELOS mass spectrometer (Thermo-Fisher) coupled to a nanoscale liquid-chromatography (LC) system (U3000 RSLC system, Thermo-Fisher). Chromatographic separation was performed on a 50-cm long reverse-phase capillary column (Acclaim Pepmap C18 2-μm 100 A, 75-μm i.d.) using a 1-hour gradient of acetonitrile. Two kinds of experiments were performed. First, a typical survey method was used for the fragmentation of the peptides (MS/MS), in which full MS scans were acquired at 60,000 resolution (FWMH) using the Orbitrap analyzer (on a mass-to-charge ratio (m/z) range of 300 to 2000) while the collision-induced dissociation (CID) spectra (MS/MS) for the eight most intense ions were recorded in the linear LTQ trap. Targeted methods were further used in which only peptides with a m/z ratio similar to selected Cxcr1 and Cxcr2 peptides were subjected to MS/MS fragmentation during the run (S1 Table).

Protein identification was performed with X!Tandem search engine [26]. Mass data were confronted to UniprotKB restricted to “*Mus*” taxonomy (taxon id 862507; 83192 sequences, release from March 12, 2014) [27], or searched against a custom databank made of Cxcr1 and Cxcr2 sequences. Parameters of the databank search were as follows: mass accuracy for peptides was set at ± 5ppm, cysteines were modified by iodoacetamide (this modification was introduced during the sample preparation), and possible oxidation of methionines and sulfation of tyrosines were considered. The mass spectrometry proteomics data have been deposited to the ProteomeXchange Consortium (http://proteomecentral.proteomexchange.org) via the PRIDE partner repository [28] with the dataset identifier PXD002244.

### Statistical analysis

Statistical analyses were performed using non-parametric Mann-Whitney test or Kruskal-Wallis test followed by Dunn’s post test for comparisons of respectively two or four groups. The Spearman test was used to analyze correlation between *Cxcr1* and *Cxcr2* mRNA levels including all data, for the four strains. All experiments were performed at least three times.

## Results

**Fig 1:**
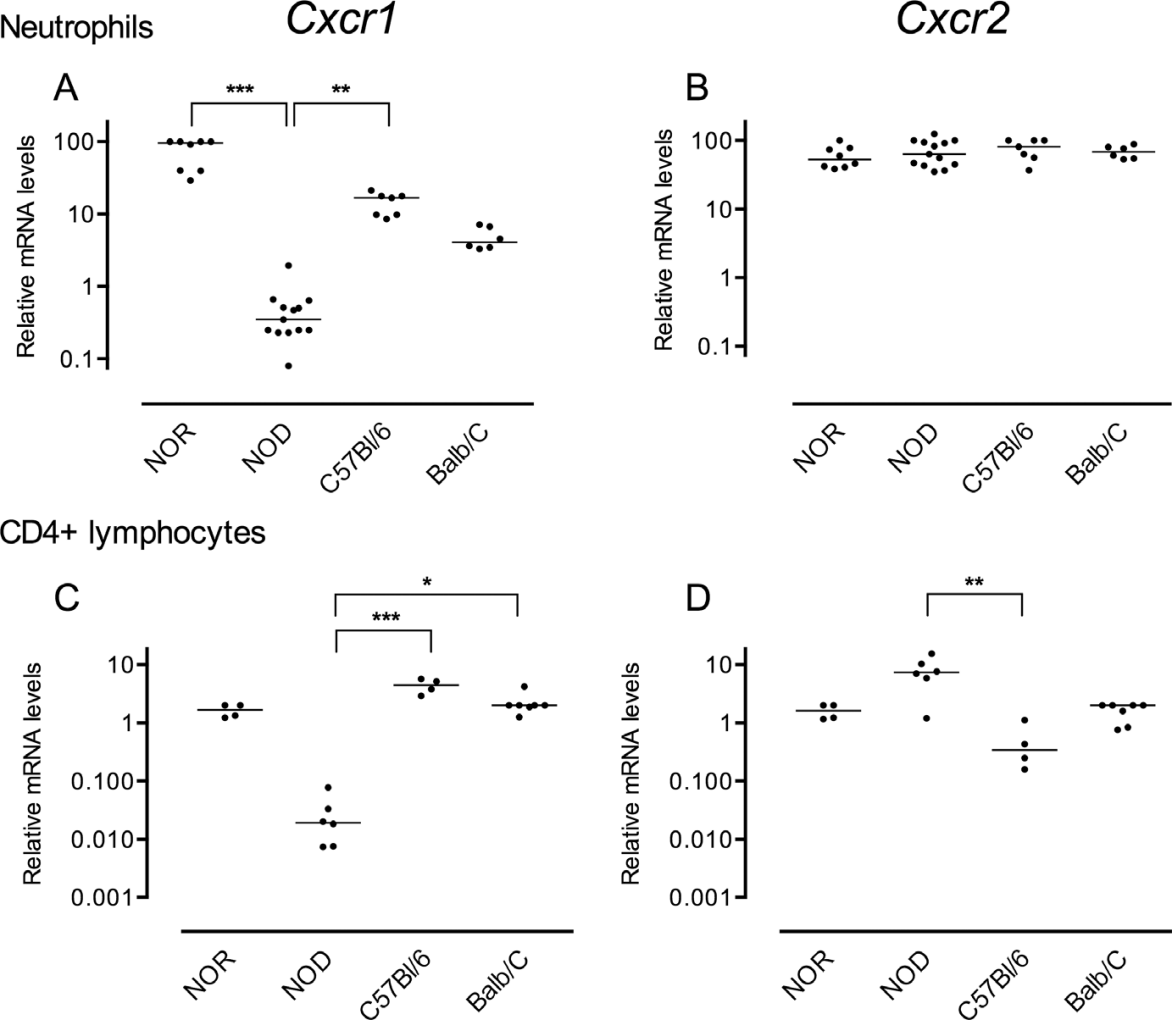
*Cxcr1* (A and C) and *Cxcr2* (B and D) mRNA levels in mouse neutrophils (A and B) and CD4+ lymphocytes (C and D). Relative *Cxcr1* and *Cxcr2* mRNA expression in neutrophils and CD4+ lymphocytes isolated from different mouse strains. Data were analyzed with the relative quantification standard curve method and expression data were normalized to *ß-actin* and *Rpb1* housekeeping genes. Each dot represents an independent sample; lines are medians. A, B: pooled data of five independent experiments are shown (*Cxcr1*- Kruskal-Wallis test: p<0.0001; Dunn’s post test NOD versus NOR, p<0.001; NOD versus C57Bl/6J: p<0.05. *Cxcr2*- Kruskal-Wallis test, ns). C, D: pooled data of three independent experiments are shown (*Cxcr1*- Kruskal-Wallis test: p<0.001; Dunn’s post test NOD versus C57Bl/6J, p<0.001; NOD versus Balb/C: p<0.05. *Cxcr2*- Kruskal-Wallis test: p<0.005; Dunn’s post test NOD versus C57Bl/6J, p<0.01).

In human, CXCR1 and CXCR2 are mainly expressed by polymorphonuclear neutrophils, thus we first analyzed *Cxcr1* and *Cxcr2* mRNA expression in murine neutrophils isolated from bone marrow. As shown in Fig 1A and 1B, we found decreased mRNA levels of *Cxcr1* in neutrophils isolated from NOD mouse compared to control strains (p<0.0001), while the NOR mouse exhibited higher levels of *Cxcr1* mRNA. In contrast, neutrophils isolated from the four murine strains exhibited comparable levels of *Cxcr2* mRNA (p = 0.5239). We also detected decreased *Cxcr1* mRNA expression in CD4+ lymphocytes isolated from NOD mouse spleen compared to control strains (Fig 1C; p<0.001) although the *Cxcr1* mRNA levels were overall lower than those measured in neutrophils. Interestingly, we observed increased *Cxcr2* mRNA levels in NOD derived CD4+ lymphocytes compared to control strains (Fig 1D; p<0.005) with an inverse correlation between *Cxcr1* and *Cxcr2* mRNA levels in CD4+ cells (Spearman test, r = -0.6239, p = 0.0025). These results suggested that the deficit in *Cxcr1* mRNA expression in NOD mouse might be genetically-controlled.

**Fig 2:**
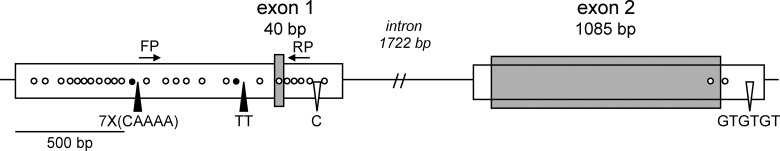
Mouse *Cxcr1* gene as reported in Ensembl genome browser. Grey boxes represent the 2 exons and white boxes the sequenced genomic regions. The polymorphims identified in NOD mouse are indicated: SNP, dot; deletion, down-pointing triangle; insertion, up-pointing triangle. Novel polymorphisms are shown with black symbols. White symbols represent already described polymorphisms (from left to right: rs32767494; rs49301700; rs255314163; rs240607825; rs222186838; rs251549700; rs237458647; rs212560494; rs241775200; rs217171203; rs233281069; rs213387676; rs264822252; rs261409730; rs240728664; rs262420321; rs236155847; rs49084468; rs259028728; rs49118666; rs31820133; rs244224727; rs49235955; rs236363650; rs262149174; rs243077358; rs52511704; rs215564316).

**Fig 3:**
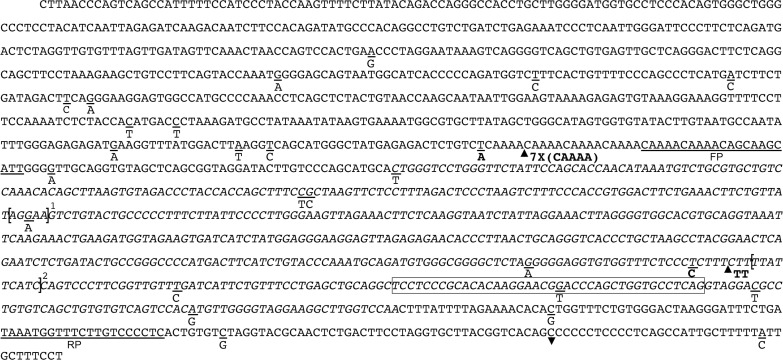
Mouse *Cxcr1* promoter region sequence. Box represents the first exon and nucleotides in italics the putative Genomatix promoter. The FP and RP primer sequences mentioned in Fig 2 are underlined. Polymorphisms found in NOD DNA sequence are indicated as follows: the SNPs are underlined while insertions are indicated by black up-pointing triangles and deletion, by black down-pointing triangle. Novel polymorphisms are shown in bold. Sequences in brackets represent putative *Spi1* box^1^ and transcription initiation site^2^. The NOD sequence has been deposited in the Genbank database under identifier KT156754.

The *Cxcr1* gene, located on chromosome 1 (reverse strand), is made of two exons of respectively 40 bp and 1085 bp, separated by a 1.7 kb intron (Fig 2). The entire open reading frame is inside the second exon and encodes a putative 351 amino acid protein. *Cxcr1* is mapped to the *idd5*.*2* susceptibility locus for type 1 diabetes. *mCxcr1* gene and 5’-flanking region sequence, prepared from NOR mouse DNA, was 100% homologous to the nucleotide sequence available in public database and is further on referred as wild-type sequence. In contrast, we discovered four novel variations (two SNPs and two insertions) in NOD *Cxcr1* genomic sequence (Figs 2 and 3). The 7X(CAAAA) insertion is a new variation of the polymorphism registered as rs218348544. We also found 26 already described variations, including 19 variations not mentioned as validated in the SNP database (http://www.ncbi.nlm.nih.gov/SNP/). The only SNP found in the coding sequence did not affect protein sequence since it encodes a synonymous amino acid.

**Fig 4:**
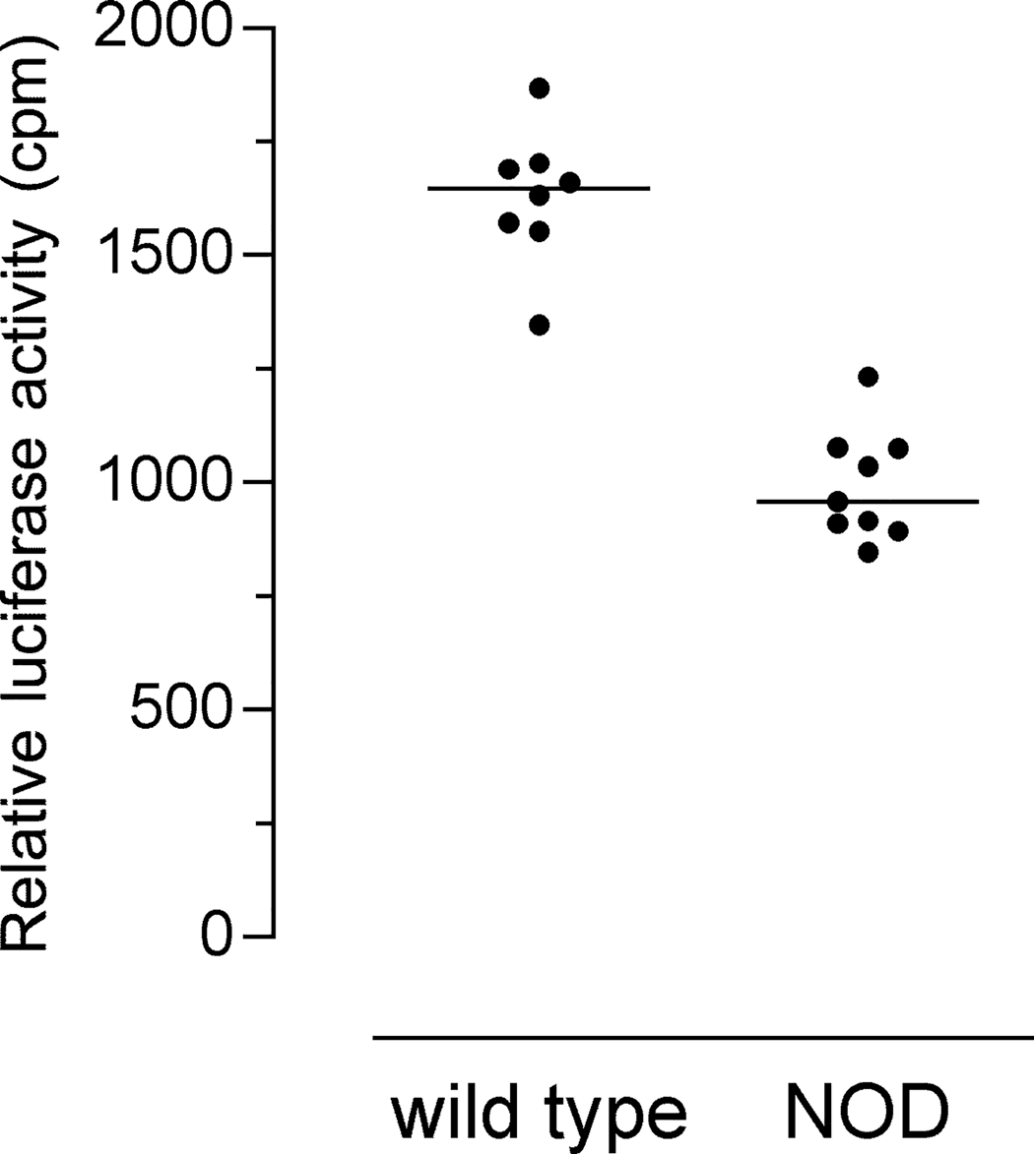
Relative Luciferase activity of wild-type and NOD promoters. The promoter region delineated by FP and RP primers (as shown in Fig 2) was used. After transfection in MH-S cells, the Luciferase activity of the experimental Firefly vector was measured and normalized to the one obtained with the control Renilla vector. Each dot represents an independent transfection in 3 different experiments (line: median; Mann-Whitney test, p<0.0001).

Of note, polymorphisms were concentrated into the 5’-flanking region and around the first non coding exon. This region matches the 601 bp-long putative promoter region which spreads from 500 bp upstream from the first exon (40 bp-long) to 61 bp downstream (Figs 2 and 3). A TTATTCATC sequence, described as the transcription initiation site in human *CXCR1* sequence [29] is logically found 54 bp upstream from the first exon. Polymorphisms may affect transcription factors binding efficiency, thus we assess whether polymorphisms of the NOD *Cxcr1* promoter affect its transcriptional efficiency. We cloned wild-type and NOD putative promoters into Firefly luciferase reporter plasmids. After nucleofection into MH-S cells, we observed that the NOD mouse sequence drove lower luciferase activity than the wild-type one (Fig 4). We did not examine further the role of each polymorphism. It is noteworthy that the rs262420321 SNP placed 353 pb upstream from the first exon, is located in a putative site of binding for PU-1-related *Spi1* transcription factor. A similar PU-1 site was shown to be essential for human *CXCR1* expression [30]. Thus, our results showed that DNA promoter sequence affects mRNA levels possibly through impaired binding of transcription factors to the mutated promoter of *mCxcr1* gene in NOD mice.

In an attempt to detect Cxcr1 expression at the protein level, we first used antibody-based methods such as western blot or flow cytometry, but we did not find any commercially available reliable antibody against mouse Cxcr1. We next performed proteomic experiments on whole-membrane fractions isolated from mouse NOR neutrophils. Two kinds of mass spectrometry methods were used for the analysis, either targeted or non-targeted towards Cxcr1 (see Material and Methods section). The non-targeted approach resulted in the identification of nearly 1000 proteins with E-values better than 10^−4^, including Cxcr2. In the example shown in S2 Table, Cxcr2 was identified with 3 peptides, a logE value of -18, and at rank 449 among 996 proteins, ranked by score. Cxcr2 was also unequivocally identified by the targeted approach. However, no reliable identification of Cxcr1 was achieved, either through the non-targeted approach or the targeted one. We also tried two methods to reduce membrane protein sample complexity before mass spectrometry analysis (1) membrane sample fractionation according to protein size, using migration onto a 4%-20% SDS-PAGE gel, and further processing of multiple slices corresponding to 25kDa to 100kDa proteins; and (2) enrichment of the microsomal fraction in integral membrane proteins by sodium carbonate treatment. None of these methods improved Cxcr1 protein identification (data not shown).

## Discussion

The first descriptions of the murine *Cxcr1* gene occurred almost 10 years ago. In 2005, Fu *et al*. published the cloning and characterization of the murine ortholog of human *CXCR1*, while open reading frame of *mCxcr1* with identical sequences were simultaneously released by other laboratories [31].

*Cxcr1* mRNAs were previously found by RT-PCR in different murine tissues such as lung and bone marrow and in isolated immune cells [31, 32]. Here, using quantitative RT-PCR, we described differences in *Cxcr1* mRNA levels in neutrophils isolated from different strains of mice although the levels of *Cxcr2* transcripts in these cells were similar. We also found decreased *Cxcr1* expression in CD4+ lymphocytes isolated from NOD mouse concomitant with increased *Cxcr2* expression, which suggests the existence of a compensatory mechanism. Mouse Cxcr1 protein is referred to Q810W6 in Uniprot Database. Despite numerous efforts, we could not find evidence of the Cxcr1 protein in our samples neither using commercially available antibodies nor by mass spectrometry.

### Anti-Cxcr1 antibodies reliability

To our knowledge, only three publications mentioned endogeneous murine Cxcr1 protein expression and all of them used antibody-based methods (immunofluorescence, flow cytometry and Western blot) [33–35]. As far as the first one is concerned, we agree with R. Ransohoff’s letter to the editor about his comments on anti-CXCR1 antibody, which fits for Bishayi’study too [36]. Indeed, some polyclonal antibodies raised against human CXCR1 are sold as cross-reactive with mouse Cxcr1 protein. However looking at the sequences of immunogenic peptides, and because of protein sequence homology in mCxcr2 and mCxcr1 compared to hCXCR1, the specificity of binding of these antibodies to mouse Cxcr1 protein remains quite questionable. We have also tested unsuccessfully a polyclonal anti mouse Cxcr1 antibody which was finally removed from the supplier catalogue.

Such problems of antibody specificity are often encountered especially when target proteins belong to the 7-TM receptor family because of structural similarities of all 7-TM receptors and their high degree of homology between subtypes within the same family [37]. Antibody validation is not always done using correct positive controls such as detection of endogeneous protein (and not only purified-recombinant protein or protein expressed in transfected cells) as well as negative controls (using preimmune serum if applicable, showing the absence of signal in target-deficient tissue whenever available…) and it becomes urgent to develop reliable criteria for antibody validation.

### Mass spectrometry for Cxcr1 protein identification

The first evidence of mouse Cxcr1 expression at the protein level was provided by Lubec and coworkers who identified Cxcr1 protein in hippocampal protein extracts into a 2D electrophoresis-spot using MS analysis [38]. Other evidence was also reported in several proteomic assays available in the PRIDE database [39]. Browsing the PRIDE archives for the Uniprot accession number of mouse Cxcr1 (#Q810W6) results in five studies reporting Cxcr1 detection (accessed on january 19, 2015). However, these assays were not primarily focused on Cxcr1 identification and they do not bring a convincing evidence that the protein was unequivocally identified. In fact, a few numbers of peptides were retrieved (one peptide per study, except for the PRD000458 one in which three peptides were detected), and in all cases, a poor correlation of their MS/MS spectra with the expected peptide sequences was obtained. Likewise, until recently, none of the MS studies performed on human neutrophil proteomes had reported CXCR1 expression [40–44]. In 2012, Bleijerveld *et al*. appended the PRIDE database following an in-depth analysis of human neutrophil proteome by 2-dimensional LC MS/MS [45]. In this study, more than 3000 proteins were identified, with relative abundances spanning over 5 orders of magnitude. Human CXCR1 (UniprotKB #P25024) and CXCR2 (UniprotKB #P25025) were respectively identified with 3 and 2 peptides. Their similar relative abundance suggests that both have close expression levels in human neutrophils.

In the present work, we attempted to detect mouse Cxcr1 in membrane-enriched neutrophil protein extracts by performing mass spectrometry experiments. Noteworthy, we demonstrated unambiguously Cxcr2 while no convincing indication of Cxcr1 presence was found. The number of identified proteins in our study is lower than that identified by Bleijverld *et al*. which might arise from differences in the experimental and analytical protocols as well as in the criteria used to validate protein hits (only proteins having a E-value lower than 10^−4^ were considered in our study). However, the comprehensiveness of our study should have been sufficient to identify Cxcr1 if its expression level was close to that of Cxcr2. The fact that it was not detected in our samples suggests that, if present, Cxcr1 is expressed at much lower abundance in mouse neutrophils than Cxcr2. Searching for Cxcr1 protein in different kinds of cells or tissues, might be an alternative to prove its existence. Assuming that *Cxcr1* mRNA is translated to protein, we questioned whether it drives a biological process or not.

### Murine CXCR1: functional or not?

According to Fan *et al*., mCxcr1 is a functional receptor for mouse Cxcl5 and human IL-8 since Ba/F cells transfected with Cxcr1 acquire the ability to migrate towards those two chemokines [46]. In these experiments, Cxcl1 and Cxcl2, the two ligands of the well-described Cxcr2 receptor, were found to be inactive to stimulate [^35^S]GTPγs exchange in mCxcr1 membranes ruling out, at the moment, the possibility that mCxcr1 might ligate those two chemokines. In agreement with these results, *Cxcr2* knockout abrogated mouse neutrophils ability to migrate towards Cxcl1 and Cxcl2 *in vitro* [47–49].

In contrast, according to Moepps *et al*., who used transfection experiments in sf9 cell line, mCxcr1 is not activated neither by hIL-8, nor mCxcl5 [32]. Also *Cxcr2*-/- neutrophils did migrate neither towards IL-8 nor towards mCxcl5 *in vitro* [47, 50]. It should be noted that Cxcl3 and Cxcl7 have not been tested in these experiments, although both have been suggested to bind CXCR1 in human [15, 51]. Using *Cxcr2* conditional deletion, Ransohoff and coworkers also reported the absence of recruited *Cxcr2*-deficient neutrophils in peritoneum following thioglycollate i.p. injection [52]. Actually, different studies in mouse suggest a fundamental and non redundant role of Cxcr2 in inflammation [4]. For example, *in vivo* experiments in *Cxcr2*-deficient mice or mice treated with blocking antibodies convincingly demonstrated the involvement of Cxcr2 in host susceptibility to infections [53–56] and in experimentally-induced autoimmune diseases [57, 58] again arguing against a strong role for mCxcr1 in mouse neutrophils.

To appreciate Cxcr1 role in mouse, additional studies in *Cxcr1*-deficient mouse/cell and *Cxcr2*-deficient mouse/cell are required. We have tried to block Cxcr2 signaling in wild-type neutrophils using anti-Cxcr2 neutralizing antibodies, but in *in vitro* migration experiments, we did not find any experimental conditions that allowed complete inhibition of Cxcr2-induced migration though Cxcl2, known as a Cxcr2 ligand.

Also, it has to be noted that identification of Cxcr1 ligands might not be yet exhaustive, which makes past functional studies incomplete. Even ELR+ chemokines, such as Cxcl1 and Cxcl2, not regarded now as mCxcr1 ligands, might activate mCxcr1 through non-conventional ß-arrestin-mediated signaling as described for other 7-TM chemokine receptors [59].

### CXCR1 involvement in type 1 diabetes?

Here, we described polymorphisms in the *Cxcr1* gene promoter region in NOD mouse, compared to the wild-type sequence and showed that some of these polymorphisms all together modulated mRNA expression levels. We now make the assumption that the effects of *Cxcr1* gene polymorphisms might be added to the one of *Slc11a1* gene resulting to T1D pathogenesis. At the moment, with regard to autoimmune diabetes in the NOD model, *Slc11a1* gene is the candidate susceptibility gene in the *idd5*.*2* locus. In this respect, *Slc11a1*-deficient female NOD mouse were recently shown to be protected from diabetes [19]. Regrettably, the authors did not specify whether their experiment has been conducted in Specific Pathogen Free facilities, which appears essential to draw a definitive conclusion about the direct role of Slc11a1 in diabetes resistance. Indeed, mouse health status is known to greatly influence diabetes incidence in NOD colony. Bacteria, fungi and parasite infections protect NOD mice from autoimmunity and delay T1D [60–64]. Thus, *Slc11a1*-deficient mice, which are susceptible to intracellular pathogen infections, might also become resistant to diabetes as a consequence of an infection.

In 2014, in the NOD model, Lehuen and coworkers reported the presence of Cxcr2+ neutrophils in pancreatic islets of young NOD mice, whereas none were found in Balb/C and C57BL/6J mice pancreatic islets [65, 66]. Neutrophil recruitment was assigned to ß cells and infiltrating macrophages which were shown to produce Cxcl1 and Cxcl2 chemokines in NOD pancreatic islets. The early blockade of neutrophil chemotaxis into the islets of neonatal NOD mice using the CXCR1/2 SB225002 antagonist decreased diabetes incidence in NOD mice [66]. Recently, Citro *et al*. demonstrated that oral administration of ladarixin, which is a potent blocker of both CXCR1 and CXCR2, even reverted diabetes in recent onset diabetic NOD mice, thus identifying the Cxcr1/2 pathway as “master regulators” of diabetes pathogenesis [67].

In our study, we found lower amounts of *Cxcr1* mRNA in neutrophils and CD4+ lymphocytes isolated from NOD mice as compared to diabetes-resistant mice. We speculate that low Cxcr1 expression in NOD mouse contributes to diabetes pathogenesis. One hypothesis is that mCxcr1 acts as a scavenger receptor to clear chemokines from circulation and tissues. Although mCxcr1 can not be considered as a member of the atypical chemokine receptor (ACKR) subgroup, whose function is fine-tuning of their respective ligands concentration and gradient [68, 69] other classical chemokine receptors have been shown to act as scavenger receptors [70]. Thus, in NOD mouse, low/no expression of mCxcr1 in pancreatic islets might make chemokines available to induce recruitment and/or activation of Cxcr2+ cells. Alternatively, impaired Cxcr1 to Cxcr2 expression ratio in CD4+ lymphocytes in NOD mouse might promote recruitment of pathogenic rather than regulatory T lymphocytes in pancreatic islets. Deciphering whether correlation of low Cxcr1 expression with type 1 diabetes in mouse is involved in disease pathogenesis still requires further experiments such as proving Cxcr1 protein expression in healthy pancreatic islets or analyzing the consequences of *Cxcr1* gene deletion in NOR mice.

In human, CXCR1 receptor is already known to be involved in different diseases. *CXCR1* mRNA and protein expression was found to be reduced on acute pyelonephritis prone patient-neutrophils in association with polymorphisms in *CXCR1* gene [71, 72]. In ulcerative colitis (UC), increased epithelial CXCR1 protein expression was found [73] and a meta-analysis afterwards identified *CXCR1* and *CXCR2* as additional UC risk loci [74]. Interestingly, *CXCR1* coding variants were also described that might be involved in asthma and COPD pathogenesis [75]. Hence, studying *CXCR1* gene polymorphisms in T1D-prone patients might provide new insights into the mechanisms that determine T1D pathogenesis. Also, understanding CXCR1 role in T1D could help to decide whether to include CXCR1/2 inhibitors or specific CXCR2 antagonists in future clinical trials.

## Supporting Information

S1 Table List of targeted Cxcr1 and Cxcr2 peptides. In targeted MS/MS experiments, only peptides of similar m/z ratio than the expected ones were fragmented by collision-induced dissociation. Three peptides corresponding to Cxcr2 sequences were introduced into the list as control. Cxcr1 peptides were defined from *in silico* digestion by trypsin according to its available sequence in UniprotKB using the PeptideMass search engine (http://web.expasy.org/peptide_mass/). The mass-to-charge values for the targeted ions were presumably defined by considering the possible charge states of the peptides. Underlined M and C respectively represent oxidized methionines and alkylated cysteines.
(DOCX)

S2 Table List of mouse neutrophil membrane-enriched proteins identified by LC-MS/MS analysis. Identification was performed with the X!Tandem program against the UniprotKB restricted to the taxonomy “*Mus*”. Proteins and peptides were filtered according to their E-value; cutoff for peptides was set at 10^−2^, and for proteins at 10^−4^. With these filters, the False Discovery Rate for proteins was found to be less than 0.2%. Protein grouping was performed using the X!Tandem pipeline (http://pappso.inra.fr/bioinfo/xtandempipeline). Redundancy was removed and proteins sharing sets of common peptides reduced to a single group. Data have been deposited to the ProteomeXchange Consortium via the Pride partner repository under the dataset identifier PXD002244. For each protein, the table includes its accession number and description as referenced in UniprotKB, its molecular weight, its rank according to E-value (as determined by the X!Tandem software), the number of specific peptides identified above the threshold score of 10^−2^, the sequence coverage, and finally, an indication of its relative abundance as evaluated from the spectral counting (PAI value).
(XLSX)
