# Supplementary material for: Type 1 Diabetes Prone NOD Mice Have Diminished Cxcr1 mRNA Expression in Polymorphonuclear Neutrophils and CD4+ T Lymphocytes
Source: PLoS One. 2015 Jul 31;10(7):e0134365. doi: 10.1371/journal.pone.0134365 (PMC4521788; doi:10.1371/journal.pone.0134365)
Supplement: S1 Table — Three peptides corresponding to Cxcr2 sequences were introduced into the list as control. Cxcr1 peptides were defined from in silico digestion by trypsin according to its available sequence in UniprotKB using the PeptideMass search engine (http://web.expasy.org/peptide_mass/). The mass-to-charge values for the targeted ions were presumably defined by considering the possible charge states of the peptides. Underlined M and C respectively represent oxidized methionines and alkylated cysteines. (DOCX) [file pone.0134365.s001.docx]

| **Protein** | **Peptide sequence** | **Charge state** | **m/z** |
| --- | --- | --- | --- |
| Cxcr1 | MAEAEYFIWTNPEGDFEK | 2 | 1088.980 |
| Cxcr1 | MAEAEYFIWTNPEGDFEK | 2 | 1096.978 |
| Cxcr1 | AEAEYFIWTNPEGDFEK | 2 | 1023.460 |
| Cxcr1 | VPITNR | 2 | 350.211 |
| Cxcr1 | RVPITNR | 3 | 285.844 |
| Cxcr1 | QAYKPFR | 2 | 455.251 |
| Cxcr1 | THMRQK | 2 | 408.711 |
| Cxcr1 | THMRQK | 2 | 400.713 |
| Cxcr1 | ILANHGLVR | 2 | 496.804 |
| Cxcr1 | ILANHGLVR | 3 | 331.538 |
| Cxcr1 | ILANHGLVRK | 2 | 560.851 |
| Cxcr1 | ILANHGLVRK | 3 | 374.237 |
| Cxcr1 | KEVLTHR | 3 | 294.843 |
| Cxcr1 | EVLTHR | 2 | 377.714 |
| Cxcr1 | EVLTHRR | 3 | 304.179 |
| Cxcr1 | VAFHTSLTAIY | 2 | 611.827 |
| Cxcr1 | EFNFFSGILLLACISVDR | 2 | 1051.043 |
| Cxcr1 | YLAIVHATR | 2 | 522.303 |
| Cxcr1 | SGTVCYEVLGEATTDFR | 2 | 952.938 |
| Cxcr1 | TLARK | 2 | 294.695 |
| Cxcr1 | TLARKR | 3 | 248.833 |
| Cxcr1 | GLSHIFGFLLPLLTMLVCYGLTLR | 2 | 1367.763 |
| Cxcr1 | GLSHIFGFLLPLLTMLVCYGLTLR | 3 | 912.178 |
| Cxcr2 | IMATYGLVSK | 2 | 549.797 |
| Cxcr2 | EGRPSFVSSSSANTSTTL | 2 | 914.440 |
| Cxcr2 | VNLSTLVCYEDVGNNTSR | 2 | 1020.797 |
